# UK primary care research: we need more researchers of Black heritage

**DOI:** 10.3399/bjgp24X739173

**Published:** 2024-08-30

**Authors:** Adwoa F Agyemang-Benneh, Christian Mallen, Joe Kai, Ralph K Akyea

**Affiliations:** Centre for Primary Care and Health Systems Research, School of Health Sciences, University of Manchester, Manchester.; National Institute for Health and Care Research (NIHR) Professor of General Practice and Director for the NIHR School for Primary Care Research, Faculty of Medicine and Health Sciences, Keele University, Keele.; Centre for Academic Primary Care, School of Medicine, University of Nottingham, Nottingham.; Centre for Academic Primary Care, School of Medicine, University of Nottingham, Nottingham.

Primary care in the UK has evolved significantly to enhance accessibility, continuity, and comprehensiveness of care, addressing the growing complexity of patient needs. Its characteristic GP-centred model has transitioned to a more integrated and multidisciplinary approach, including preventive care, chronic disease management, and mental health support. As the first port of call, the goal of any nation’s primary care system is to be responsive to the prevailing health needs of its local communities. Globally, primary care is crucial to ensuring accessible, non-fragmented, and patient-centred care.^1^ Researchers in the field of primary care are therefore responsible for providing sufficient evidence to achieve this goal, through rigorous research underpinned with a contextual lens.

Primary care researchers add to knowledge of current advances in medical research but move a step further to ensure its applicability to the communities and individuals for whom these advances have been designed.^2^ To achieve this, research teams must have adequately qualified academics, and more importantly, adequate representation of populations whose circumstances are being explored.

Individuals who identify as Black (that is, descended from Black African, Black Caribbean, and other Black origin) represent approximately 4.0% of the total population of England and Wales, but London for instance, has around 13.3% of its population identifying as Black, indicating significant regional variations.^3^ However, despite the growing proportion of Black students entering undergraduate and postgraduate education, the representation of Black individuals in UK higher education remains disproportionately low. Many universities have contributed substantially to an increasingly inclusive student population over the past decade, but there are still few Black applicants accepted into PhD programmes.^4^ Torjesen suggests that this could possibly be because Black students are more likely to receive comparatively lower undergraduate degrees or that stereotyping exists unconsciously in the university examination and recruitment system.^4^

Either way, these barriers contribute to Black academics representing only 1.4% of the total academic workforce in the 2020/2021 academic year (Higher Education Statistics Agency [HESA]). Furthermore, there were only 160 out of a total of 22 855 professors in the UK who identified as Black as of January 2022, according to the HESA database. Again, Arday argues that Black staff are less likely than their White counterparts to progress to senior management positions or professorships, be employed on permanent contracts, or be placed on higher salary bands.^5^

There is a paucity of ethnicity-aggregated data for primary care researchers in the UK. We suspect that due to the limited number of primary care researchers of Black heritage across the UK, such an aggregation of data might potentially lead to easily identifiable individuals. Nonetheless, it is important to explore diversity in primary care research because of its effect on the health outcomes of populations. Diversity is multifaceted, with important primary (for example race and gender) and secondary (for example, education and religion) elements that impact the effectiveness of any workforce.^6^ It matters in research for many reasons, beyond ensuring a representative participant population, and diversity in research teams is important as researchers are key in interpreting findings and translating these into practice. Each person’s lens and unique perspective when conducting research helps to focus on what is considered relevant to current healthcare narratives. When a research team consists of individuals of varied ethnicities, a broader research focus may be facilitated with a drive to investigate a wider array of health issues that affect different populations in different ways. Additionally, diversity helps to acknowledge and consider societal and health disparities that may be overlooked by a more homogeneous team.

Where under-represented populations are part of a study, it is essential for participants to interact with researchers from these or similar populations to improve the ease of communication between researchers and participants.^7^ Researchers from under-represented backgrounds also help to build and sustain trust from participants and help for acceptability of interventions as well.^8^

Diversity reduces unconscious bias, which occurs even in the most well-intended of studies. For survival, our ancestors learned to quickly classify encounters as friendly or dangerous before deciding on their interaction, and we have adopted this evolutionary trait to quickly categorise our encounters as well.^9^ In the same way, it is easy to categorise study participants and data into stereotypes, without considering diversity within and across ethnic groups. Diversity in research teams lends itself to a more inclusive study design and implementation, ensuring a robust study whose findings will potentially be more accessible in practice.

We should do better in primary care research because we argue for the centrality of primary care to improve health outcomes for all populations. It does not help to classify all minority ethnic groups under one umbrella term because it masks inequalities within these groups and provides a skewed picture of specific health inequalities.^10^ How Black groups view and experience primary care may be radically different from other ethnic groups and must be emphasised to contextually address ethnicity-based health inequalities, design culturally sensitive interventions, and acknowledge the implications of being racialised as Black in the UK.^11^ Ensuring that our research teams adequately represent the populations whose health outcomes we seek to improve can help us move closer to our purpose. At the very least it can help us understand more clearly what we are trying to improve.

The Board of the National Institute for Health and Care Research School for Primary Care has commissioned a project to explore the under-representation of Black researchers in primary care research. This is a commendable first step to unpack the barriers that Black researchers may be facing in primary care research. Other institutions such as the Wellcome Trust and the Royal Society of Chemistry are also exploring under-representation through similar research projects, to understand the barriers and opportunities that influence the career progression of researchers and scientists of Black heritage.^12^^,^^13^

The Russell Group’s report on strengthening UK research provides a toolkit on how funders and universities could be more inclusive in research careers, work experience, and respectful environments.^14^^,^^15^ One key recommendation worth emphasising is the need to provide mentors for researchers from under-represented communities. Additionally, researchers from under-represented groups, especially Black heritage, serve as accessible role models who provide an essential source of encouragement for younger generations thinking of this career pathway.

Going further, leaders in primary care research could co-develop programmes that seek to even the playing field and lend support to outstanding individuals of Black heritage to start and continue on the academic trajectory within primary care research. As a first step, this can be done through a series of workshops and focus groups that identify best practices of representation across the UK and beyond, and how these can be operationalised in the UK context.

In conclusion, there is a need for more researchers of Black heritage, a need to attract and retain promising individuals from this demographic, and a need to do right by the communities we serve in primary care, by ensuring diversity and inclusivity in research teams.

## References

[1] World Health Organization (2018). Declaration of Astana. https://www.who.int/publications/i/item/WHO-HIS-SDS-2018.61.

[2] Hobbs R (2019). Is primary care research important and relevant to GPs?. Br J Gen Pract.

[3] Office for National Statistics (2024). Population of England and Wales.

[4] Torjesen I (2021). How to shrink the gap that holds black scientists back. Nature.

[5] Arday J (2022). The black professoriate: assessing the landscape within British higher education. On Education Journal for Research and Debate.

[6] Hubbard EE (2004). The Manager’s Pocket Guide to Diversity Management.

[7] Kai J, Hedges C (1999). Minority ethnic community participation in needs assessment and service development in primary care: perceptions of Pakistani and Bangladeshi people about psychological distress. Health Expect.

[8] Salway S, Chowbey P, Such E, Ferguson B (2015). Researching health inequalities with community researchers: practical, methodological and ethical challenges of an ‘inclusive’ research approach. Res Involv Engagem.

[9] Marcelin JR, Siraj DS, Victor R (2019). The impact of unconscious bias in healthcare: how to recognize and mitigate it. J Infect Dis.

[10] Race Disparity Unit (2022). Why we no longer use the term ‘BAME’ in government. https://equalities.blog.gov.uk/2022/04/07/why-we-no-longer-use-the-term-bame-in-government.

[11] Ojo-Aromokudu O, Suffel A, Bell S, Mounier-Jack S (2023). Views and experiences of primary care among Black communities in the United Kingdom: a qualitative systematic review. Ethn Health.

[12] Baptista D (2023). We’re exploring targeted funding to support Black researchers — here’s how you can help. https://wellcome.org/news/were-exploring-targeted-funding-black-researchers.

[13] Royal Society of Chemistry Black representation in UK academic chemistry. https://www.rsc.org/policy-evidence-campaigns/inclusion-diversity/surveys-reports-campaigns/black-representation-in-uk-academic-chemistry.

[14] Gottlieb G, Smith S, Cole J, Clarke A Realising our potential: backing talent and strengthening UK research culture and environment.

[15] Gottlieb G, Smith S, Cole J, Clarke A Research Culture and Environment Toolkit.

